# Purification and Characterization of BmooAi: A New Toxin from *Bothrops moojeni* Snake Venom That Inhibits Platelet Aggregation

**DOI:** 10.1155/2014/920942

**Published:** 2014-05-29

**Authors:** Mayara Ribeiro de Queiroz, Carla Cristine N. Mamede, Nadia Cristina G. de Morais, Kelly Cortes Fonseca, Bruna Barbosa de Sousa, Thaís M. Migliorini, Déborah Fernanda C. Pereira, Leonilda Stanziola, Leonardo A. Calderon, Rodrigo Simões-Silva, Andreimar Martins Soares, Fábio de Oliveira

**Affiliations:** ^1^Instituto de Genética e Bioquímica, Universidade Federal de Uberlândia, 38400-902 Uberlândia, MG, Brazil; ^2^Instituto Nacional de Ciência e Tecnologia em Nano-Biofarmacêutica (N-Biofar), 31270-901 Belo Horizonte, MG, Brazil; ^3^Instituto de Ciências Biomédicas, Universidade Federal de Uberlândia, 38400-902 Uberlândia, MG, Brazil; ^4^Centro de Estudos de Biomoléculas Aplicadas à Saúde (CEBio), Fundação Oswaldo Cruz Rondônia (Fiocruz Rondônia) e Núcleo de Saúde, Universidade Federal de Rondônia (UNIR), 76812-245 Porto Velho, RO, Brazil

## Abstract

In this paper, we describe the purification/characterization of BmooAi, a new toxin from *Bothrops moojeni* that inhibits platelet aggregation. The purification of BmooAi was carried out through three chromatographic steps (ion-exchange on a DEAE-Sephacel column, molecular exclusion on a Sephadex G-75 column, and reverse-phase HPLC chromatography on a C2/C18 column). BmooAi was homogeneous by SDS-PAGE and shown to be a single-chain protein of 15,000 Da. BmooAi was analysed by MALDI-TOF Spectrometry and revealed two major components with molecular masses 7824.4 and 7409.2 as well as a trace of protein with a molecular mass of 15,237.4 Da. Sequencing of BmooAi by Edman degradation showed two amino acid sequences: IRDFDPLTNAPENTA and ETEEGAEEGTQ, which revealed no homology to any known toxin from snake venom. BmooAi showed a rather specific inhibitory effect on platelet aggregation induced by collagen, adenosine diphosphate, or epinephrine in human platelet-rich plasma in a dose-dependent manner, whereas it had little or no effect on platelet aggregation induced by ristocetin. The effect on platelet aggregation induced by BmooAi remained active even when heated to 100°C. BmooAi could be of medical interest as a new tool for the development of novel therapeutic agents for the prevention and treatment of thrombotic disorders.

## 1. Introduction

Snake venoms are a complex mixture of various proteins, enzymes, and other substances with toxic properties. Among the complex pool of proteins (more than 90% of the dry weight) are included enzymes such as acetylcholinesterases, aminotransferases, phosphoesterases, ADPases, phospholipases, hyaluronidases, L-amino acid oxidases (LAAOs), and proteases (metalloproteinases and serinoproteases) [1–5]. Protein C activators, growth factors (NGF, VEGF), lectins, precursors of bioactive peptides, von Willebrand factor binding proteins, disintegrins, and bradykinin potentiators are some representatives of nonenzymatic components from snake venom [2, 6].

Snake venoms contain a wide variety of nonenzymatic and enzymatic components that have very specific effects on platelet aggregation [7]. Some phospholipases A_2_ (PLA_2_) can affect platelet function usually due to phospholipid hydrolysis and the formation of metabolites of arachidonic acid [8–10]. Serinoproteases can activate platelet aggregation directly by proteolytic cleavage of protease-activated receptors (PARs) or by binding to the GPIb receptor [6, 9, 11]. Several snake venom metalloproteinases (SVMPs) have been shown to interfere with platelet function through specific structural or enzymatic effects on platelet receptors or their ligands [6–8]. The effects on platelet aggregation caused by LAAOs are generally related to platelet exposure to hydrogen peroxide which is generated by the enzymatic activity of the toxin [9, 12, 13]. Disintegrins are usually nonenzymatic inhibitors of platelet aggregation, which typically inhibit *β*_1_, *β*_3_, and *β*_5_ integrins. A common feature of disintegrins is the presence of the arginine-glycine-aspartate tripeptide sequence (RGD) or a homologous, non-RGD sequence, in their integrin-binding sites. Due to their binding to the fibrinogen receptor GPIIb/IIIa (*α*_IIb_ *β*_3_), disintegrins inhibit platelet aggregation induced by a wide range of agonists [14–17]. Snake venom C-type lectins are also able to affect platelet function by binding to Von Willebrand factor (vWF) or receptors such as GPIb, *α*_2_ *β*_1_, and GPVI [18, 19]. In contrast, inhibitory effects of 5′-nucleotidases on platelet aggregation probably occur via catalytic activity that causes the degradation of ADP, a platelet aggregation agonist [6].

In this paper, we describe the purification of BmooAi from* B. moojeni* venom and its characterization as a new toxin that inhibits platelet aggregation.

## 2. Materials and Methods

### 2.1. Material

Desiccated* B. moojeni *venom was purchased from Bioagents Serpentarium (Batatais, SP, Brazil). Acetonitrile, acrylamide, ammonium bicarbonate, ammonium persulphate, bromophenol blue, bovine fibrinogen, glycine,  *β*-mercaptoethanol, *N*, *N*′-methylene-*bis*-acrylamide, sodium dodecyl sulphate (SDS), *N*, *N*, *N*′, *N*′-tetramethylethylenediamine (TEMED), trifluoroacetic acid, and Tris were purchased from Sigma Chemical Co. (St. Louis, MO, USA). Molecular mass markers for electrophoresis and all chromatographic media (DEAE-Sephacel, Sephadex G-75, and C2/C18 columns) were purchased from GE Healthcare Technologies (Uppsala, Sweden). All the agonists used in the platelet aggregation assays (collagen, adenosine diphosphate, epinephrine, and ristocetin) were purchased from Helena Laboratories (Beaumont, Texas, USA). All other reagents used were of analytical grade.

### 2.2. Blood Collection

Human blood was obtained from volunteer-donors. The experiments reported here follow the guidelines established by the Human Research Ethics Committees of Universidade Federal de Uberlândia (CEP/UFU), Minas Gerais, Brazil (Protocol number 055/11).

### 2.3. Purification of BmooAi

BmooAi was first purified using the methodology previously described [20] with modifications. Crude venom from the* B. moojeni* snake (400 mg) was dissolved in 50 mmol/L ammonium bicarbonate buffer (pH = 7.8) and clarified by centrifugation at 10,000 ×g for 10 minutes. The supernatant solution was fractionated in a DEAE-Sephacel column (2.5 × 20.0 cm) previously equilibrated with 50 mmol/L ammonium bicarbonate (AMBIC), pH = 7.8. Elution was carried out at a flow rate of 20 mL/h with a concentration gradient (50 mmol/L–0.6 mol/L) of the same buffer. Fractions with 3.0 mL/tube were collected and their absorbance was recorded at a wavelength of 280 nm. Fractions corresponding to peak DS4 were pooled, lyophilized, dissolved in 50 mmol/L AMBIC, pH 7.8, and then applied to a Sephadex G-75 column (1.0 × 100.0 cm) previously equilibrated with the same buffer. The flow rate was 20 mL/hour, fractions of 3.0 mL were collected, and their absorbance was recorded at a wavelength of 280 nm. Fraction DS4G2, showing antiplatelet activity, was pooled, lyophilized, dissolved in solvent A (0.1% trifluoroacetic acid), and then subjected to reverse-phase chromatography in a C2/C18 column (4.6 × 100 mm) using the ÄKTApurifier HPLC system. The column was equilibrated with solvent A and eluted applying a concentration gradient toward solvent B (0.1% trifluoroacetic acid containing 80% acetonitrile) from 0 to 100% for column volume at a flow rate of 0.5 mL/min at room temperature. Absorbance was monitored at wavelengths of 214 and 280 nm and 1 mL fractions were collected.

### 2.4. Estimation of Protein Concentration

The protein concentration of the fractions was determined using a UV absorption method that calculates concentration from absorbance at 214 nm, using a BioSpec-mini spectrophotometer (Shimadzu Biotech, Japan).

### 2.5. Electrophoretic Analysis

Electrophoresis using polyacrylamide gel (SDS-PAGE) was performed as previously described [21] using 14% gels. Electrophoresis was carried out at 20 mA/gel in Tris-glycine buffer, pH 8.3, containing 0.01% SDS. The molecular mass standard proteins used were phosphorylase b (97,000), bovine serum albumin (66,000), ovalbumin (45,000), carbonic anhydrase (30,000), soybean trypsin inhibitor (20,000), and *α*-lactalbumin (14,000). Gels were stained with Coomassie blue R-250, 0.2% (w/v).

### 2.6. MALDI-TOF Mass Spectrometry Analysis

The molecular mass of BmooAi was analyzed by MALDI-TOF mass spectrometry using a 4800 MALDI TOF/TOF mass spectrometer (Applied Biosystems, Foster City, California, USA) as previously described [22] with modifications.

### 2.7. N-Terminal Sequence Determination

The N-terminal sequence of BmooAi was determined by Edman degradation [23] performed on an automated sequencer (Procise model 494, Applied Biosystems). The identity of the primary sequence of BmooAi was compared with other proteins using BLAST (Basic Local Alignment Search) (http://blast.ncbi.nlm.nih.gov/Blast.cgi).

### 2.8. Peptide Synthesis of N-Terminal Sequences

Both the Ile-Arg-Asp-Phe-Asp-Pro-Leu-Thr-Asn-Ala-Pro-Glu-Asn-Thr-Ala and Glu-Thr-Glu-Glu-Gly-Ala-Glu-Glu-Gly-Thr-Gln sequences were synthesized using GenScript (Piscataway, New Jersey, USA).

### 2.9. Proteolytic Activity on Fibrinogen

Fibrinogenolytic activity was assayed as previously described [24] with modifications. Fibrinogen (1.5 mg/mL) and samples (5 *μ*g) were mixed 1 : 100 (w/w) and the mixture was incubated at 37°C for 120 min. The reaction was stopped by the addition of an equal volume of a denaturing buffer containing 2% sodium dodecyl sulphate (SDS) and 10% *β*-mercaptoethanol. Reaction products were analyzed using 14% (w/v) SDS-PAGE.

### 2.10. Platelet Aggregation

Platelet aggregation assays were performed in human platelet-rich plasma (PRP) and measured using an automated 4-channel Aggregometer (AggRAM version 1.1, Helena Laboratories, USA). Human blood collected in sodium citrate (3.2%) was centrifuged at 100 ×g for 12 min at room temperature to obtain PRP. Platelet-poor plasma (PPP) was obtained from the residue by centrifugation of citrated blood at 1,000 ×g for 15 min. Assays were carried out using 200 *μ*L of PRP maintained at 37°C under continuous stirring in siliconized glass cuvettes. Aggregation was triggered with collagen (10 *μ*g/mL), ADP (20 *μ*M), ristocetin (1.5 mg/mL), or epinephrine (300 *μ*M) after the incubation of platelets with different doses of BmooAi (0.6, 1.0, and 1.4 *μ*g). One hundred percent (100%) aggregation was expressed as the percentage absorbance relative to PPP aggregation. Control experiments were performed using only platelet agonists. All experiments were carried out in triplicate.

## 3. Results and Discussion

**Figure 1:**
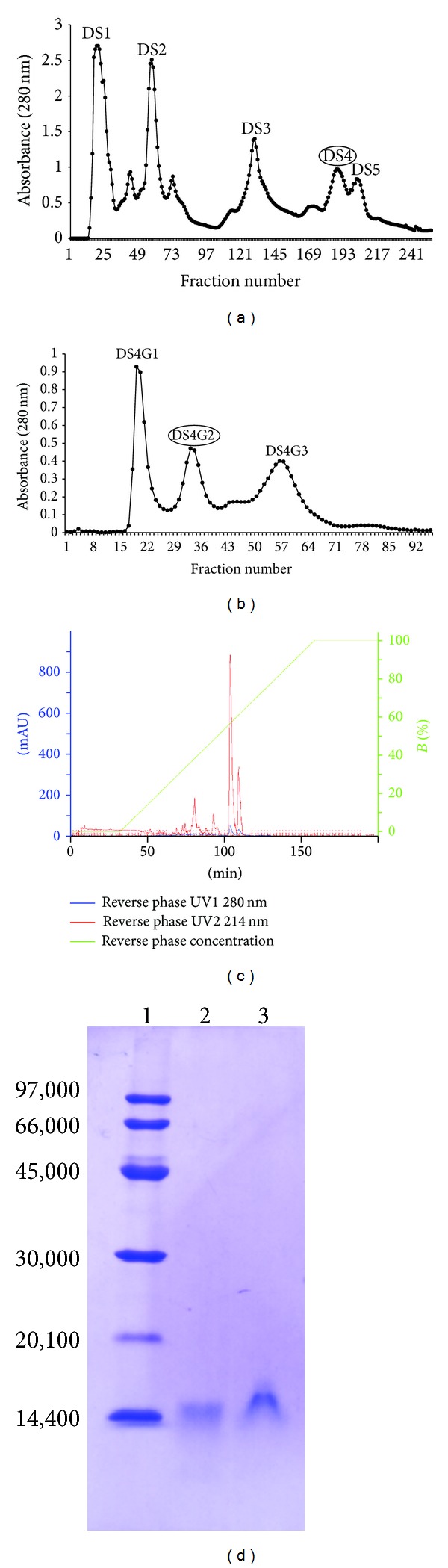
Sequential purification steps of BmooAi from* Bothrops moojeni *venom. (a) Ion-exchange chromatography on a DEAE-Sephacel column: crude venom (400 mg) was applied to the column (2.5 × 20 cm) and elution was carried out at a flow rate of 20 mL/h with ammonium bicarbonate gradient buffer (50 mmol/L–0.6 mol/L). Fractions of 3.0 mL/tube were collected and their absorbance read at 280 nm. (b) Molecular exclusion on a Sephadex G-75 column: the active fraction (DS4) was applied to the column and eluted with 50 mmol/L ammonium bicarbonate buffer at pH 7.8 with a flow rate of 20 mL/hour. (c) Reverse-phase HPLC chromatography on a 2.0 × 2.5 cm C2/C18 column (GE Health Care), equilibrated with solvent A (0.1% trifluoroacetic acid) and eluted with a concentration gradient of solvent B (80% acetonitrile and 0.1% trifluoroacetic acid) from 0 to 100% at a flow rate of 0.5 mL/min at room temperature. (d) SDS-PAGE in a 14% (w/v) gel. Lanes: 1: standard proteins; 2: reduced BmooAi fraction; 3: nonreduced BmooAi fraction. The gel was stained with Coomassie blue R-250.

In this study, we describe the purification and partial characterization of a new toxin from* B. moojeni *venom that inhibits platelet aggregation. The fractionation of the* B. moojeni* venom was carried out by three chromatographic steps involving ion-exchange chromatography on a DEAE-Sepharose column, molecular exclusion chromatography on a Sephadex G-75 column, and reverse-phase HPLC chromatography on a C2/C18 column. The fractionation of* B. moojeni* venom by ion-exchange chromatography resulted in five major protein fractions named DS1 through DS5 (Figure 1(a)). Fraction DS4 was further fractionated by size exclusion chromatography (Sephadex G-75) and three fractions were collected (DS4G1 through DS4G3) (Figure 1(b)). All fractions were tested on collagen-, ristocetin-, epinephrine-, or ADP-induced platelet aggregation in human plasma. Fraction DS4G2 (80 *μ*g) inhibited around 85% of collagen-induced and around 75% of ADP-induced platelet aggregation; it was also able to degrade both the A*α* and B*β* chains of bovine fibrinogen (results not shown). Even when preincubated at 100°C, fraction DS4G2 maintained its inhibitory effect on platelet aggregation, but lost its fibrinogenolytic activity (results not shown). These results suggest that the effect on aggregation induced by fraction DS4G2 is not dependent on enzymatic action, since the proteins present in this fraction were denatured by a high temperature. Fraction DS4G2 was lyophilized and subjected to reverse-phase HPLC chromatography on a C2C18 column (Figure 1(c)). This procedure resulted in four major protein fractions at 214 nm but only two at 280 nm. These results suggest that the first two peaks at 214 nm are composed of proteins poor in aromatic amino acids. The first peak at 214 nm was also analyzed by SDS-PAGE and showed a single polypeptide chain around 15 kDa (Figure 1(d)). This peak was able to interfere with platelet aggregation and was named BmooAi (*Bothrops moojeni *platelet aggregation inhibitor). BmooAi showed no fibrinogenolytic activity.

BmooAi seems to have low expression in* B. moojeni* snake venom, since it represented ~0.005% (w/w) of the initial crude venom. It is not advantageous when compared to the overall yield of other protein molecules with inhibitory effects on platelet aggregation such as atroxlysin-I from* B. atrox *[25] or* Bl*-LAAO from* B. leucurus *[26], which represent around 5.1 and 3.7% (w/w) of their crude venoms, respectively. In this study, we had to repeat the purification steps several times in order to obtain sufficient material for an initial characterization of this new toxin. For this reason, BmooAi concentration determinations were performed by a UV absorption method; thus, there was no sample waste from using traditional methods to determine protein concentration. The low recovery of BmooAi likely discouraged other researchers from investigating this protein, mainly due to the challenges of its purification. In spite of the disadvantages, BmooAi has high antiplatelet activity that may contribute significantly to the overall effects of envenomation by* B. moojeni.*

**Figure 2:**
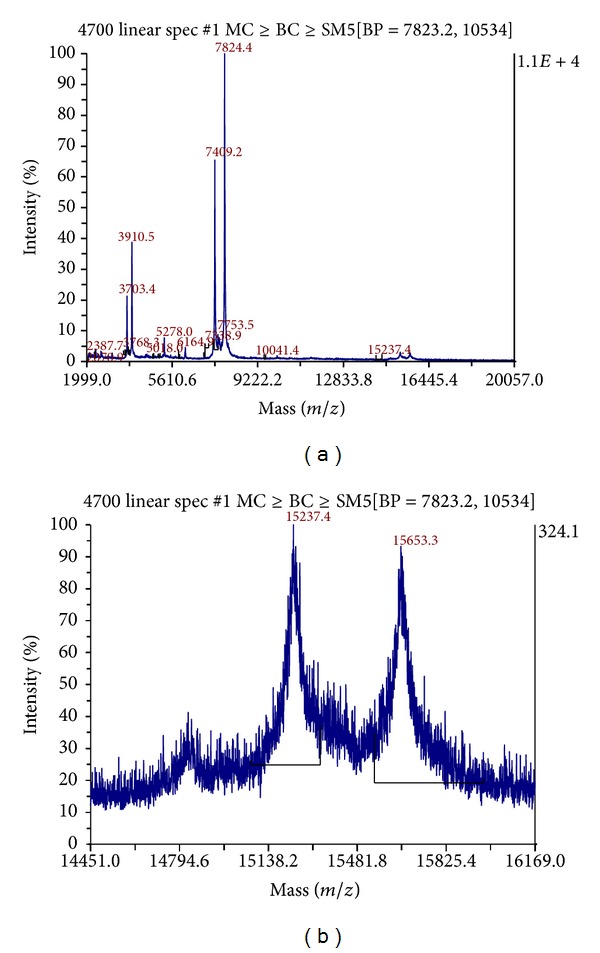
Mass determination of BmooAi by MALDI-TOF mass spectrometry. (a) The fraction consisted of two major components with molecular masses (M + H) of 7824.4 and 7409.2. These two compounds are also seen as doubly charged ions (*M*/*Z* = 3910.5 and 3703.4, resp.). (b) Expansion of the area around 15 kDa to better visualize the presence of a trace of this protein.

Mass spectrometry analysis of BmooAi indicated two major components with molecular masses (M + H) 7824.4 and 7409.2 (Figure 2(a)). These two compounds are also seen as doubly charged ions (*M*/*Z* = 3910.5 and 3703.4, resp.).  Figure 2(b) shows traces of a protein with a molecular mass (15237.4) similar to that found via SDS-PAGE (Figure 1(d)). Based on the analysis by SDS-PAGE (single band) and reverse-phase chromatography (symmetric peak), we suggest that BmooAi is a unique protein that undergoes autolysis/proteolysis, releasing two peptides of molecular mass around 7.5 kDa. Indeed, some snake venom toxins can undergo proteolysis/autolysis under nonphysiological conditions* in vitro*, such as in the presence of reducing agents, alkaline pH, or low calcium concentration [27]. Additionally, the presence of two peptides composed of different amino acids corroborates the suggestion that they originate from the autolysis/hydrolysis of BmooAi, since peptides that differ in hydrophobicity should elute in different peaks in reverse-phase chromatography.

BmooAi was subjected to N-terminal sequencing by Edman degradation and revealed two amino-acid sequences: IRDFDPLTNAPENTA and ETEEGAEEGTQ. Both N-terminal sequences were submitted to BLAST but neither shared homology with other snake venom protein. Interestingly, the primary sequence of BmooAi has the sequence APEN in the same position (residues 10–13) occupied by the identical sequence in Insularin, a disintegrin from* B. insularis *venom that inhibits platelet aggregation induced by ADP [28]. This finding deserves attention, but more studies are needed to elucidate the importance of this sequence for inhibition of platelet aggregation.

Platelets play an essential role in hemostasis. Alterations in normal platelet function are involved in various thrombotic and cardiovascular disorders [29, 30]. Modulation of platelet activation and aggregation are currently applied to treat and prevent cardiovascular disorders and stroke [6, 29, 31, 32].

**Figure 3:**
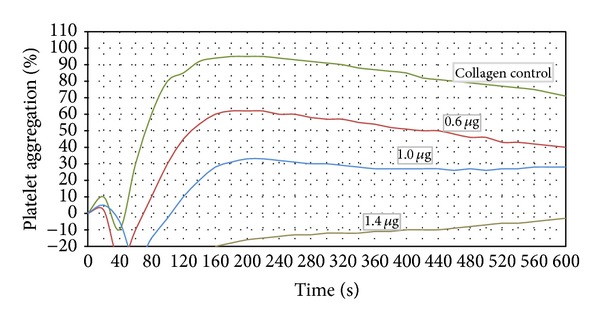
Effect of BmooAi (0.6, 1.0, and 1.4 *μ*g) on collagen-induced platelet aggregation. Human PRP was preincubated with the indicated doses of BmooAi for 8 min at 37°C before adding collagen (10 *μ*g/mL). Platelet aggregation was recorded for 10 min in an AggRAM platelet aggregation system with four-channel laser optics (Helena Laboratories, EUA). Results were expressed as an increase in light transmission, where PPP represents the maximum response (100%). Control experiments were performed in the absence of BmooAi.

**Figure 4:**
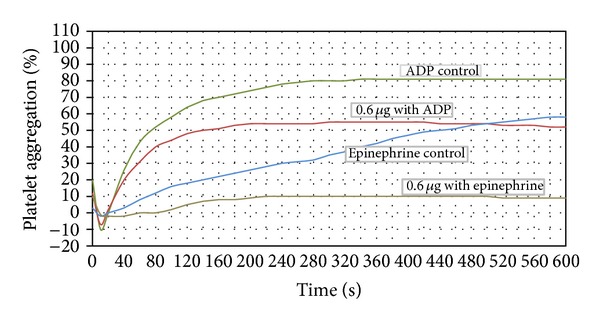
Effect of BmooAi (0.6 *μ*g) on ADP- and epinephrine-induced platelet aggregation. Human PRP was preincubated with the indicated dose of BmooAi for 8 min at 37°C before adding ADP (20 *μ*mol/L) or epinephrine (300 *μ*mol/L). Platelet aggregation was recorded for 10 min in an AggRAM platelet aggregation system with four-channel laser optics (Helena Laboratories, EUA). Results are expressed as an increase in light transmission, where PPP represents the maximum response (100%). Control experiments were performed in the absence of BmooAi.

In this study, we characterized the interference of BmooAi with agonist-induced platelet aggregation (collagen, ADP, epinephrine, and ristocetin). Our results showed that BmooAi inhibited collagen-induced (10 *μ*g/mL) platelet aggregation in a concentration-dependent manner. Complete inhibition of collagen-induced platelet aggregation was obtained with only 1.4 *μ*g of BmooAi (Figure 3). Even after heating to 100°C, BmooAi maintained its inhibitory activity, supporting the hypothesis that its inhibition of platelet aggregation is nonenzymatic in nature (data not shown). The assays of inhibition of ADP-, epinephrine-, and ristocetin-induced platelet aggregation were performed using a dose of 0.6 *μ*g, due to the low amount obtained from purification. BmooAi inhibited over 80% of epinephrine-induced (300 *μ*M) platelet aggregation and around 30% of ADP-induced aggregation (20 *μ*M) (Figure 4). Under the same conditions, BmooAi did not show any inhibitory effect on ristocetin-induced (1 mg/mL) aggregation.

Platelet aggregation is characterized by the accumulation of platelets into a hemostatic plug. The GPIIb/IIIa receptor plays a central role in linking activated platelets. Independent of the initial stimulus, blocking integrin *α*_IIb_ *β*_3_ prevents platelet aggregation and subsequent thrombus formation by preventing binding to fibrinogen. The participation of integrin *α*_IIb_ *β*_3_ in platelet aggregation, whatever the initiating event or agonist, justifies the interest in the therapeutic blockade of this receptor, since all routes of platelet activation converge on to this final common pathway [7, 31, 33–35]. Here, we show that BmooAi can inhibit ADP-, epinephrine-, and collagen-induced platelet aggregation at low concentrations, which suggests that this toxin may act on integrin *α*_IIb_ *β*_3_. On the other hand, the lack of an inhibitory effect on ristocetin-induced platelet aggregation suggests that BmooAi does not interfere with von Willebrand factor.

Disintegrins are a family of cysteine-rich low-molecular-mass polypeptides (40–100 amino acids) present in viperid venoms that are usually nonenzymatic inhibitors of platelet aggregation [6, 14, 15, 17]. They typically have an RGD sequence that binds to integrin *α*_IIb_ *β*_3_ and other integrins inhibiting their functions [15, 28, 36]. The RGD sequence presents inhibitory activity on platelet aggregation induced by several agonists [6]. However, the Gly position can be occupied by other individual amino acid residues or even by two residues in conformationally restrained peptides and still retain integrin-binding activity [36]. In the venom of the same snake species there are disintegrins that exhibit a conserved RGD-motif and disintegrins with variable non-RGD sequences, such MLD, MGD, VGD, KGD, WGD, or RTS/KTS [16, 17]. However, the short N-terminal sequence of the BmooAi, obtained in this work was not sufficient to show the presence or absence of RGD or variable non-RGD sequences.

In order to determine the influence of the N-terminal region of the peptides found, we synthesized the two peptides and evaluated their effect on aggregation. Neither synthesized peptides (50.0 *μ*g) showed any inhibitory effect on ADP-, epinephrine-, ristocetin-, or collagen-induced platelet aggregation. These results suggest that the antiplatelet action of BmooAi depends not only on its N-terminal but also on other regions formed by adjacent amino acids and the C-terminal may be essential for its activity [16, 17, 37].

## 4. Conclusion

In conclusion, we describe a new toxin from snake venom that inhibits platelet aggregation. The reported toxin, BmooAi, has a molecular mass around 15,000 Da and showed no homology with any other snake venom toxin. BmooAi has great potential for pharmacological studies due to the low dose used to inhibit platelet aggregation and can be of medical interest as a new tool for the development of novel therapeutic agents to prevent and treat patients with thrombotic disorders.
